# Ligand-based pharmacophore modeling for the discovery of Na_V_1.7 inhibitors

**DOI:** 10.1007/s10822-026-00898-z

**Published:** 2026-08-07

**Authors:** Martina Piga, Peter Lukacs, Krisztina Pesti, György Várady, Zoltan Varga, Nace Zidar, Tihomir Tomašič

**Affiliations:** 1 Faculty of Pharmacy, University of Ljubljana, Aškerčeva cesta 7, Ljubljana, 1000 Slovenia; 2 Agricultural Institute, Centre for Agricultural Research, Martonvásár, Hungary; 3 HUN-REN Institute of Experimental Medicine, Budapest, Hungary; 4 Institute of Molecular Life Sciences, HUN-REN Research Centre for Natural Sciences, Budapest, Hungary; 5 Department of Biophysics and Cell Biology, Faculty of Medicine, University of Debrecen, Egyetem ter1, Debrecen, 4032 Hungary

**Keywords:** Na_V_1.7 inhibitors, Pharmacophore model, Selectivity, Virtual screening, Voltage-gated sodium channel

## Abstract

Voltage-gated sodium channel Na_V_1.7 is a key mediator of electrical excitability and signal transmission in peripheral nociceptors and has emerged as a highly attractive therapeutic target for the development of novel analgesic agents. However, the development of selective Na_V_1.7 inhibitors has been characterized by significant challenges, with repeated failures in clinical trials despite encouraging preclinical data. In this study, we developed and validated a series of ligand-based pharmacophore models (LBPMs) that can be useful for the discovery of novel Na_V_1.7 inhibitors with improved selectivity profiles. Using the VGSC database as our primary data source, we focused on sulfonamide-based inhibitors targeting the voltage-sensing domain IV (VSD-IV). Validation against active compounds and decoys demonstrated that most models achieved good discrimination performance with high areas under the curve (AUC) and strong enrichment factors. External validation using 22 inhibitors extracted from recent literature confirmed the models’ capability to identify novel Na_V_1.7 inhibitors. Virtual screening of 3 million commercially available compounds retrieved promising hits and known inhibitors, with molecular docking studies revealing binding modes consistent with established sulfonamide-based inhibitors. Experimental validation identified one compound with measurable selectivity for Na_V_1.7 over Na_V_1.5, providing preliminary support for the utility of the developed virtual screening workflow. In parallel, we developed Na_V_1.5 LBPMs to assess selectivity profiles and minimize potential cardiotoxic effects. Overall, our findings provide valuable computational tools and structural insights for the rational design of selective Na_V_1.7 inhibitors, offering important starting points for developing analgesics with reduced off-target effects.

**Supplementary Information:**

The online version contains supplementary material available at 10.1007/s10822-026-00898-z.

## Introduction

Voltage-gated sodium channels (Na_V_) are large transmembrane proteins that play a crucial role in the initiation and propagation of action potentials in excitable cells, including neurons, skeletal muscle, and cardiac myocytes [1, 2]. These channels facilitate the selective transport of sodium ions across the cell membrane in response to membrane depolarization [3].

The mammalian Na_V_ channel subfamily comprises nine distinct ion-conducting α-subunits (Na_V_1.1–Na_V_1.9), each encoded by a unique gene and characterized by different functional properties and specific tissue distributions [4]. Na_V_1.1, 1.2, 1.3, and 1.6 are predominantly expressed in the central nervous system (CNS), Na_V_1.4 is highly expressed in the skeletal muscles and Na_V_1.5 is the main sodium channel subtype in the cardiac muscle. Na_V_1.7, 1.8, and 1.9 are primarily localized in the peripheral nervous system (PNS) [5]. Dysfunction or specific mutations in Na_V_ channels are recognized as causes of a wide range of human pathologies, collectively known as channelopathies [5, 6]. Abnormal function of Na_V_1.1, 1.2 and 1.6 is related to the onset of genetic epilepsy. Dysfunction of Na_V_1.1 is linked to familial hemiplegic migraine, and mutations of Na_V_1.5 have been associated with various cardiac arrhythmias and inherited cardiomyopathies. Mutations in Na_V_1.4 cause some types of paralyses and Na_V_1.6 alterations have been related to the onset of neurodegenerative diseases. Na_V_1.3, 1.7, 1.8 and 1.9 mutations or aberrant function have been associated with congenital insensitivity to pain, erythromelalgia, and other pain disorders [4, 5, 7]. Given their fundamental role in cellular excitability and their involvement in various diseases, Na_V_ channels have emerged as attractive molecular targets for drug development [5].

The α-subunits of Na_V_ channels comprise four homologous domains (DI-DIV), each domain consisting of six transmembrane segments (S1–S6). N- and C-terminal residues are located on the cytoplasmic side [5]. Segments S1–S4 form the voltage-sensing domain (VSD), while S5 and S6 are mainly involved in forming the pore domain [8]. The S5–S6 transmembrane segments are bridged on the extracellular side by a P-loop, which forms the selectivity filter and defines the outer vestibule, thus representing a crucial structural motif for ion discrimination and transmembrane conductance. The voltage-sensing apparatus includes S4 segments that contain positively charged amino acid residues and represent a target site for drug binding. Under resting membrane conditions, the channels are closed: internal negative potential exerts an electrostatic force that holds the positively charged residues inwardly. Membrane depolarization triggers an outward translational displacement of the S4 segment, thereby inducing a voltage-dependent conformational change that facilitates channel opening and sodium ion conduction. Upon prolonged depolarization, the channel undergoes inactivation, resulting in the interruption of ion flux without the closure of the activation gate [5, 9]. The main α-subunit is commonly associated with one or more auxiliary β-subunits, which can contribute to both normal channel functions and additional regulatory functions [9].

Numerous Na_V_ channel blockers exhibit state-dependent binding, showing high affinity for the open or inactivated channel conformations. Other modulators preferentially bind to channels with high activation frequency, a phenomenon that is particularly advantageous for selectively targeting abnormally operating channels [5, 10]. Both natural toxins and synthetic molecules can function as pore blockers or gating modifiers of Na_V_ channels [11]. Early Na_V_ modulators, including local anesthetics, antiepileptics and antiarrhythmics, typically bind to the intracellular local anesthetic binding site [4, 5, 12]. Due to their lack of selectivity, they often cause undesirable side effects, including neurotoxicity and cardiac arrhythmias, which limit their use, especially in the treatment of chronic diseases [5]. The limited clinical utility and safety profile of non-selective Na_V_ blockers have highlighted the urgent need for isoform-specific modulation in current voltage-gated sodium channel drug discovery. However, due to the high sequence similarity between Na_V_ subtypes, it is difficult to develop inhibitors with a good selectivity profile [13]. In recent years, important progress has been made: several inhibitors have shown some selectivity for Na_V_1.3, Na_V_1.7, or Na_V_1.8 [2, 13–20]. Suzetrigine, a first-in-class Na_V_1.8 inhibitor, has recently been approved for the treatment of moderate to severe acute pain in adults [20]. Considering the repeated failures in clinical trials despite encouraging preclinical data of several earlier inhibitors, the recent approval of suzetrigine marks a pivotal breakthrough in the field [21]. This therapeutic success reinforces the validity of targeting peripheral Na_V_ isoforms for the management of chronic and neuropathic pain and validates the broader concept that selective sodium channel blockade can yield therapeutically effective and safer analgesics. It also suggests that the initial failures were not a refutation of the target class therapeutic relevance, but rather an indication of the need for more refined drug design and optimization strategies.

One of the most intensively studied structural classes of Na_V_ inhibitors in recent years are the acyl and arylsulfonamide derivatives, which bind preferentially to the voltage-sensing domain IV (VSD-IV) on the extracellular side in the inactivated conformation of the channel. The binding modes of selected potent sulfonamide inhibitors (e.g., ICA121431, GX-936, GNE-1305, GDC-0310, GNE-3565, GNE-9296, PF-05089771) bound to Na_V_-VSD-IV, particularly to Na_V_1.7-VSD-IV, were recently revealed, confirming their predicted binding poses. The negatively charged sulfonamide moiety interacts with the positively charged S4 arginine residues, while the hydrophobic tail anchors within a lipid-facing region [14, 18, 22, 23]. Most of these molecules have shown high potency, with IC_50_ values typically in the micro- to nanomolar range, and remarkable isoform selectivity. However, none have progressed to clinical use, as achieving effective analgesic activity in vivo remains challenging, partly due to suboptimal ADME and physicochemical properties [16].

Given the challenges of achieving both high potency and isoform selectivity, computational methods have gained increasing relevance as tools for guiding Na_V_-targeted drug discovery. Computer-aided drug design (CADD) offers strategic advantages in accelerating hit identification and optimizing lead compounds while significantly reducing associated costs. This strategy enables the rational design and in silico prediction of novel ligands that can bind with high affinity and, crucially, high selectivity to a desired Na_V_ isoform [11, 24].

The aim of this study was to develop a set of ligand-based pharmacophore models (LBPM) to identify new Na_V_1.7 inhibitors. Among the nine Na_V_ isoforms, genetic evidences have highlighted Na_V_1.7 (SCN9A) as one of the most attractive targets for non-opioid analgesic therapies [25–27]. Selective Na_V_1.7 inhibition can provide potent analgesia through targeted modulation of peripheral nociceptive pathways, thereby minimizing the broad systemic side effects [28]. As a source of Na_V_1.x inhibitors, we utilized the VGSC database (https://cadd.zju.edu.cn/vgsc/*)* [29] and, in later validation stages, extracted inhibitor data from the latest scientific publications. Known Na_V_1.7 inhibitors were used to construct and validate LBPMs for predicting compound activity. These models formed the basis for the virtual screening of a commercial compound library to discover new Na_V_1.7 inhibitors. In parallel, we generated LBPMs for Na_V_1.5 from known Na_V_1.5 inhibitors, enabling selectivity profiling of the virtual screening hits. Selected retrieved hits were subsequently tested in multiple concentrations to evaluate their inhibitory efficacy and subtype selectivity against Na_V_1.7 and Na_V_1.5 using a high-throughput automated patch-clamp (APC) platform. Furthermore, we aimed to determine whether the selected molecules exhibit state-dependent binding. Altogether, our findings provide valuable insights and important starting points for the rational design of new potential therapeutic agents targeting Na_V_1.7 channels, with an improved selectivity profile, that are essential to avoid potential CNS or cardiovascular side effects.

## Materials and methods

### Software

We employed LigandScout 4.5 Expert (Inte: Ligand GmbH) [30, 31] to create and validate 3D structure-based and ligand-based pharmacophore models. To generate multi-conformational compound libraries, we utilized LigandScout’s i: Con conformer generator [32, 33]. Activity profiling was carried out using InteLigand’s activity profiling algorithm, integrated into a KNIME extension and executed within the KNIME Analytics Platform [34]. This setup, supported by LigandScout Expert KNIME Extensions, facilitated virtual screening of the multi-conformational libraries against multiple 3D pharmacophore models simultaneously and produced a heat map to visualize the activity profile of each ligand.

### Compound library preparation

The data for Na_V_1.x inhibitors was extracted from the VGSC database [29] in August 2024. Within each Na_V_1.x dataset, we implemented a filtering workflow to assemble a focused small‑molecule library. First, all toxin‑ and peptide‑based molecules were eliminated. Next, we retained exclusively sulfonamide inhibitors reported to bind to the voltage‑sensing domain (VSD-IV) of the channels. Finally, to ensure dataset non‑redundancy, we removed any entries sharing identical PubChem CIDs or IUPAC nomenclature. Compounds were additionally inspected for duplicated structures arising from different naming conventions and multiple database entries. Where possible, stereochemical annotations and salt forms were standardized according to the downloaded structures. This curation reduced the initial compound count by over 50% for each isoform, yielding libraries of sulfonamide-based Na_V_ inhibitors for pharmacophore model generation: 16 Na_V_1.1 inhibitors, 22 Na_V_1.2 inhibitors, 9 Na_V_1.3 inhibitors, 7 Na_V_1.4 inhibitors, 188 Na_V_1.5 inhibitors, 20 Na_V_1.6 inhibitors, 1512 Na_V_1.7 inhibitors, 6 Na_V_1.8 inhibitors and 1 Na_V_1.9 inhibitor. A set of decoys was generated for the most potent inhibitors of each Na_V_1.x isoform using the DUDE decoys database [34]. Decoys are theoretical molecular structures that have not been experimentally tested on the respective targets. They were originally designed to evaluate docking-based virtual screening outcomes. In this study, decoys were utilized to generate receiver operating characteristic (ROC) curves, enabling a comparative assessment of the pharmacophore models’ predictive performance. A selection of the most potent compounds for each target was processed through the DUDE decoy generator, yielding 50 decoys per compound. These decoys shared similar 1D physicochemical properties with the active compounds but exhibited distinct 2D topologies.

To generate multi-conformational libraries for active, inactive, and decoy compounds, we employed i: Con using the default “FAST” settings (Timeout: 600s, RMS threshold: 0.5, energy window: 15.0, max. pool size: 4000, max. fragment build time: 30s, max. conformers: 25). Virtual screening libraries were subsequently created using LigandScout’s idbgen algorithm.

### Ligand-based pharmacophore modelling

All ligands in the actives dataset were clustered in LigandScout based on Pharmacophore RDF-code similarity using default settings. For clusters containing at least five inhibitors, ligand-based pharmacophore models were created using the merged feature pharmacophore approach, applying default parameters (omitted features: 4, partially matching features optional, threshold: 10.0%). Exclusion volume spheres were generated around the aligned ligands. For each ligand-based pharmacophore model, the ligands within the cluster were designated as the training set. All training set ligands were automatically aligned to the generated pharmacophore model to ensure consistency in molecular feature representation.

### Pharmacophore model validation

The developed structure- and ligand-based pharmacophore models were applied to screen datasets of Na_V_1.x inhibitors and decoy molecules. The scoring function was set to “Pharmacophore-fit”, and the screening mode was configured as “Match all query features” with the maximum number of omitted features set to zero. Model performance was evaluated using ROC curve analysis to assess predictive accuracy and reliability.

### Molecular docking

Ligand structures were opened in Maestro (Schrödinger, LLC, New York, NY, USA, 2024). Energy-minimized conformations of virtual screening hits were generated with the LigPrep wizard using the OPLS3 force field. The protonation states of the ligands were calculated at pH 7.4 using Epik. Molecular docking calculations were performed using Schrödinger Release 2024–1 (Schrödinger, LLC, New York, NY, USA, 2024). The cryo-EM structure of Nav1.7 in complex with the inhibitor (PDB entry: 8F0S [18]) was retrieved from the Protein Data Bank. The protein was then prepared using the Protein Preparation Wizard with default settings. The receptor grid was calculated for the ligand-binding site, and compounds were docked using the Glide XP protocol, as implemented in Schrödinger Release 2024–1 (Glide, Schrödinger, LLC, New York, NY, USA, 2024). To validate the docking protocol, the co-crystallized ligand from PDB structure 8F0S was removed and redocked into the prepared receptor. The reproduced binding pose showed an RMSD of 1.95 Å relative to the experimental structure, supporting the reliability of the docking protocol. The highest scored docking pose of each compound was used for presentation.

### Cell culture and automated patch-clamp electrophysiology

The recombinant hNa_V_1.5 and hNa_V_1.7 channel-expressing cell lines were generated and maintained as previously described [35, 36]. Electrophysiological measurements were performed using the IonFlux Mercury automated patch-clamp system (Fluxion Biosciences, Alameda, CA, USA) utilizing 64-channel ensemble recording plates. In this configuration, each recording channel represents the averaged macroscopic current of 20 trapped cells, ensuring robust and reproducible current readouts by minimizing cell-to-cell variability. The solutions and recording settings were the same as those used in our previous publications [36–38], except for the following modifications:

- The microfluidic plate was divided into eight independent zones, allowing the simultaneous testing of two different compound compositions per zone.
- To simultaneously monitor voltage-dependent activation, steady-state inactivation, and recovery from inactivation, a modified voltage protocol was employed. To test our hypothesis regarding voltage-sensor binding, the entry-into-inactivation phase of the original protocol was modified to measure the voltage-dependence of activation.

The voltage command was repeated every 5 s. State-dependent inhibition was assessed by comparing currents elicited from a holding potential (− 120 mV) representing resting-state affinity, measured at a 0 mV test pulse and currents elicited following a 50 ms depolarizing prepulse to − 100 mV to representing the inactivated state affinity, as it drives approximately 50–70% of the channels into the inactivated state (Fig. S1).

Data processing and analysis were conducted using custom-written Python scripts. The Pandas library was employed for structured data handling, multi-dimensional data transformation, and the automated import/export of Excel-based datasets. All numerical calculations, vectorized array operations, and statistical evaluations were performed using the NumPy (Numerical Python) package. Electrophysiological traces, normalized peak current distributions, and quality control (QC) parameters, inhibition levels were graphically represented using the Matplotlib visualization framework. The figures were created in Microsoft Excel.

### Compound preparation

Stock solutions were prepared in DMSO. To achieve testing concentrations up to 100 µM without exceeding a final DMSO concentration of 1% (v/v), which would cause osmotic stress, membrane destabilization, and the loss of the patch-clamp seal, 10 mM stock solutions were used.

## Results and discussion

### Preparation of compound libraries

Our approach for developing and validating pharmacophore models relied on the VGSC database as the primary source for generating relevant compound libraries [29]. The VGSC database represents an invaluable resource for Na_V_ channel-focused drug discovery, providing comprehensive, curated bioactivity and chemotype information across all nine human Na_V_1.x isoforms. Annotation of multiple ligand binding pockets enables the generation of binding site-directed libraries and experimentally determined data activity facilitates robust structure-activity relationship (SAR) analyses. The direct linkage of each entry to its primary literature ensures full traceability, and the ability to bulk download in standard formats (CSV, SDF) significantly simplifies data integration into computational modeling workflows. Standardized structural identifiers (e.g., SMILES and PubChem CIDs) support seamless chemical curation and cross-referencing. On the other hand, the database requires user caution regarding data recency and collection methodologies. The database does not include novel scaffolds published since its original release, requiring researchers to supplement data from the current literature. Moreover, although chemical identifiers are standardized, hidden duplicates may still occur due to (*i*) minor salt/conformation form differences, (*ii*) naming redundancies, especially when different alphanumeric codes are assigned to the same compound due to its multi-isoform activity. This makes de‑duplication less straightforward than it may appear.

Within each Na_V_1.x isoform dataset, we excluded toxins and peptide-based inhibitors to focus solely on small molecules. Given that VGSCs contain multiple binding sites for small molecules [39], we further refined our selection by retaining only sulfonamide-based inhibitors that specifically target the VSD of DIV. Finally, duplicate entries, identified by identical PubChem CIDs or IUPAC names, were removed to maintain a non‑redundant dataset. This filtering process significantly reduced the number of inhibitors per Na_V_1.x isoform: from 80 to 16 Na_V_1.1 inhibitors, from 304 to 22 Na_V_1.2 inhibitors, from 118 to 9 Na_V_1.3 inhibitors, from 415 to 7 Na_V_1.4 inhibitors, from 1554 to 188 Na_V_1.5 inhibitors, from 57 to 20 Na_V_1.6 inhibitors, from 3280 to 1512 Na_V_1.7 inhibitors, from 223 to 6 Na_V_1.8 inhibitors, and from 25 to 1 Na_V_1.9 inhibitor.

### Generation and validation of ligand-based pharmacophore models based on Na_V_1.7 inhibitor library

**Fig. 1:**
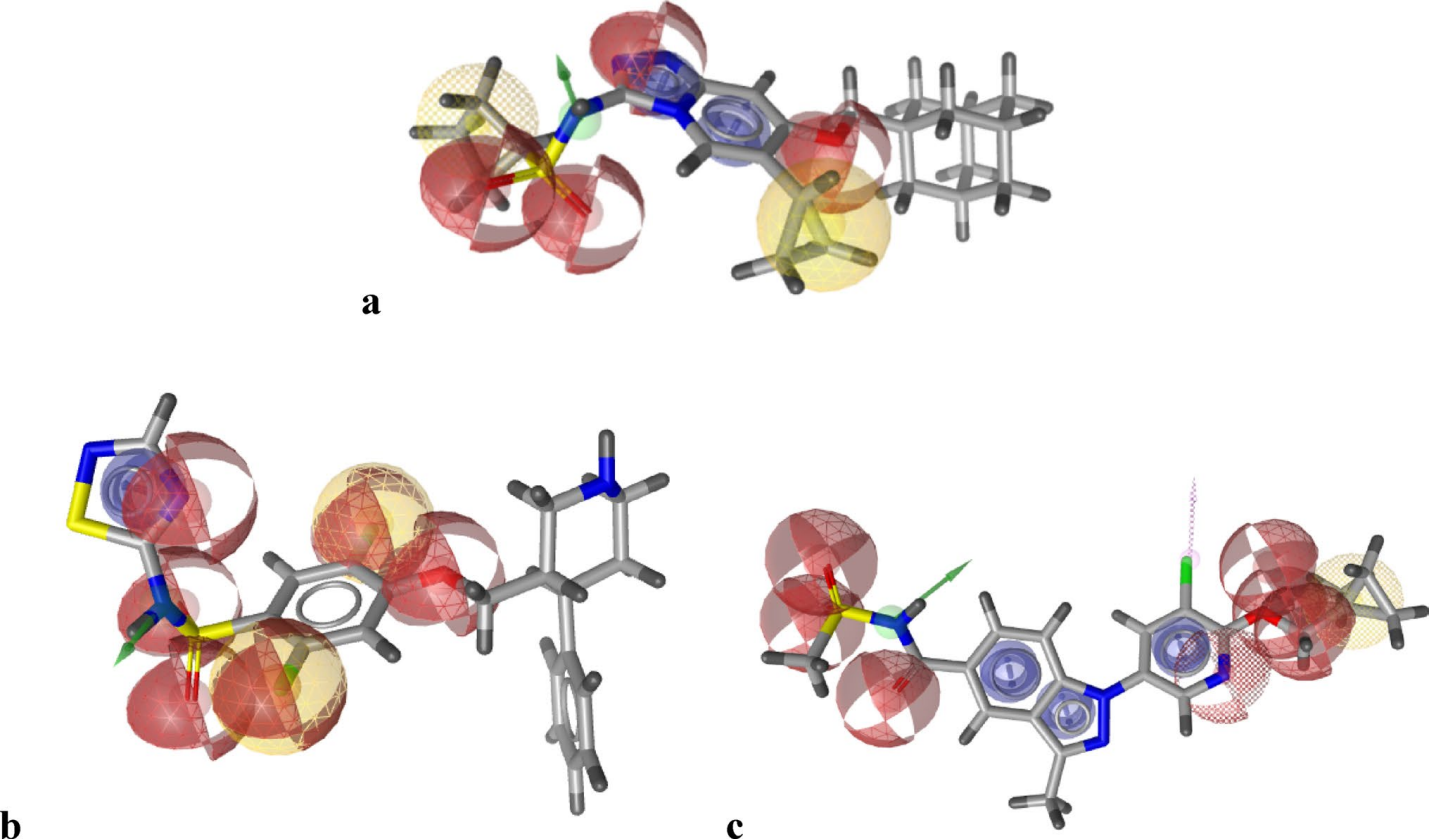
Ligand-based pharmacophore models for Na_V_1.7 inhibitors with IC_50_≤10 nM. **a** LBPM_Nav1.7_Cluster4, **b** LBPM_Nav1.7_Cluster9, **c** LBPM_Nav1.7_Cluster13. Each model is aligned with the inhibitor with the highest pharmacophore fit score. The pharmacophore features are as follows: hydrophobic (H, yellow spheres), aromatic (AR, blue disc), hydrogen bond donor (HBD, green arrow), hydrogen bond acceptor (HBA, red sphere), and halogen bond (HAL, pink arrow).

We built LBPMs from libraries filtered for Na_V_1.7 inhibitors. When the entire set of 1512 structurally diverse compounds was used, the resulting model contained only two hydrogen bond acceptor features and lacked specificity. To improve specificity and focus on the most potent inhibitors, we extracted 205 compounds with IC_50_ ≤ 10 nM and clustered them by pharmacophore radial distribution function (RDF) code similarity. Pharmacophore models were generated for clusters with at least five ligands. A minimum cluster size of five ligands was selected to ensure sufficient structural diversity while retaining adequate statistical representation for pharmacophore generation. This approach produced 13 clusters, eight of which met the size criterion. These eight clusters were subsequently used to create merged-feature LBPMs (Fig. 1 and Fig. S2).

Each model was initially validated using three Na_V_1.7 datasets comprising active compounds: one with 205 compounds (IC_50_ ≤ 10 nM), another with 605 compounds (IC_50_ ≤ 100 nM), and a complete set of 1512 Na_V_1.7 inhibitors (Table 1). **LBPM_Na**_**V**_**1.7_Cluster4**, **_Cluster5**, **_Cluster9** and **_Cluster13** were highly selective, identifying only a limited number of known Na_V_1.7 inhibitors. In contrast, **LBPM_Na**_**V**_**1.7_Cluster8**, **_Cluster10**, **_Cluster11** and **_Cluster12** were able to identify a greater number of active compounds from the dataset. Each LBPM was also screened against a combined library of active compounds and decoys generated using the DUD-E (Database of Useful Decoys - Enhanced) server [40]. To assess virtual screening performance, receiver operating characteristic (ROC) curves were generated and are shown in Fig. S3 in the Supporting Information. Most Na_V_1.7 LBPMs demonstrated good discrimination between actives and decoys, yielding high areas under the curve (AUC) and strong enrichment factors (EF), with the exception of **LBPM_Na**_**V**_**1.7_Cluster12**. To improve the specificity of the model, ligands from cluster 12 were additionally clustered using the pharmacophore alignment as a similarity measure, which resulted in eight additional clusters (**LBPM_Nav1.7_Cluster12_1** to _**Cluster12_8**). Among these, six contained more than five ligands and were used for building the LBPMs and validation as described above (Table 1, Figs. S4 and S5).

To provide a more comprehensive evaluation of model performance, additional validation metrics including sensitivity, specificity, Matthews correlation coefficient (MCC), EF1%, and EF5% were calculated. Based on the validation metrics provided in Table S1, the overall performance of the Na_V_1.7 LBPMs appears to be quite heterogeneous, reflecting the structural diversity of the training compounds and the different levels of model specificity. The early enrichment metrics (EF1% and EF5%) were relatively consistent across most models, with values ranging from 2.0 to 2.8, indicating that the pharmacophore models were generally effective in enriching active compounds among the top-ranked screening hits. Notably, several models achieved near-perfect AUC1% and AUC5% values, demonstrating excellent discrimination between active compounds and decoys in the early stages of virtual screening.

However, the models differed substantially in sensitivity and MCC, which provide a more balanced assessment of screening performance. Highly selective models such as **LBPM_Na**_**V**_**1.7_Cluster4**, **_Cluster5**, and **_Cluster13** exhibited perfect specificity (100%) but very low sensitivity (0.3–2.6%), indicating that they retrieved only a small subset of active compounds while producing virtually no false positives. In contrast, **LBPM_Na**_**V**_**1.7_Cluster11** and **_Cluster12_3** showed the best overall balance between sensitivity and specificity, achieving sensitivities of 52.9% and 67.1%, respectively, while maintaining specificity above 94%. These models also yielded the highest MCC values (0.646 and 0.657), suggesting superior overall predictive performance.

The models selected for prospective virtual screening (see below), namely **LBPM_Na**_**V**_**1.7_Cluster4**, **_Cluster9**, and **_Cluster13**, were not chosen solely on the basis of MCC or sensitivity but rather because of their high specificity and restrictive pharmacophoric constraints. Such highly selective models are particularly useful in large-scale virtual screening campaigns, where minimizing false positives is often prioritized over maximizing recall. Among these models, **LBPM_Na**_**V**_**1.7_Cluster9** displayed the most favorable balance, combining perfect specificity with substantially higher sensitivity (31.1%) and an MCC value of 0.475. Overall, the results demonstrate that different pharmacophore models offer complementary advantages, with some maximizing selectivity and others maximizing coverage of the active chemical space, thereby supporting the use of a multi-pharmacophore screening strategy.

Three high-performing Na_V_1.7 LBPMs, **LBPM_Na**_**V**_**1.7_Cluster4**, **_Cluster9**, and **_Cluster13**, are described in detail below and illustrated in Fig. 1, while additional models are shown in Figs. S1 and S3 (Supplementary Information). **LBPM_Na**_**V**_**1.7_Cluster4** (Fig. 1a) features four hydrogen bond acceptors (HBA, red spheres), one hydrogen bond donor (HBD, green arrow), one aromatic ring (AR, blue disc), and two hydrophobic features (H, yellow spheres), one of them optional (dotted sphere) and the other essential (solid sphere). **LBPM_Na**_**V**_**1.7_Cluster9** (Fig. 1b) includes six HBAs, one HBD, two hydrophobic features, and one AR feature, making it more feature rich. **LBPM_Na**_**V**_**1.7_Cluster13** (Fig. 1c) contains seven HBAs (one optional), one HBD, three AR features, one optional H, and one optional halogen bond feature (HAL, dotted pink arrow).

**Table 1:** Hit rates of virtual screening using Na_V_1.7 pharmacophore models

	Na_V_1.7 IC_50_ ≤ 10 nM	Na_V_1.7 IC_50_ ≤ 100 nM	Na_V_1.7 all	Virtual screening hits
	205 inhibitors	605 inhibitors	1512 inhibitors	3 million compounds
LBPM_Nav1.7_Cluster4	4 hits	10 hits	21 hits	13 hits
LBPM_Nav1.7_Cluster5	3 hits	4 hits	5 hits	0 hits
LBPM_Nav1.7_Cluster8	107 hits	262 hits	796 hits	519 hits
LBPM_Nav1.7_Cluster9	10 hits	28 hits	88 hits	1 hit
LBPM_Nav1.7_Cluster10	17 hits	54 hits	125 hits	22,955 hits
LBPM_Nav1.7_Cluster11	56 hits	164 hits	800 hits	627 hits
LBPM_Nav1.7_Cluster12	190 hits	539 hits	1343 hits	/
LBPM_Nav1.7_Cluster12_3	67 hits	207 hits	1014 hits	49,379 hits
LBPM_Nav1.7_Cluster12_4	12 hits	31 hits	53 hits	0 hits
LBPM_Nav1.7_Cluster12_5	19 hits	36 hits	73 hits	0 hits
LBPM_Nav1.7_Cluster12_6	58 hits	107 hits	149 hits	1 hit
LBPM_Nav1.7_Cluster12_7	40 hits	71 hits	118 hits	0 hits
LBPM_Nav1.7_Cluster12_8	58 hits	183 hits	471 hits	1 hit
LBPM_Nav1.7_Cluster13	5 hits	19 hits	39 hits	1 hit

To assess whether these LBPMs for Na_V_1.7 can also be used for selectivity prediction, the set of LBPMs was screened against the inhibitor libraries of other Na_V_1.x isoforms, together including 269 inhibitors. In total, 135 Na_V_1.x inhibitors were identified as hits: 10 for Na_V_1.1, 15 for Na_V_1.2, 6 for Na_V_1.3, 3 for Na_V_1.4, 84 for Na_V_1.5, 15 for Na_V_1.6 and 2 for Na_V_1.8. This shows that the specificity of the LBPMs for Na_V_1.7 is limited. However, most of the retrieved molecules exhibited significant inhibitory activity on Na_V_1.7 channels, with IC_50_ values in the micromolar range. The total number of compounds retrieved by each LBPM varied substantially, highlighting their diverse recognition patterns and the structural heterogeneity of the training compounds from which they were derived. Notably, **LBPM_Na**_**V**_**1.7_Cluster4**, **_Cluster9**, and **_Cluster10** emerged as the most selective models, retrieving only 3, 1, 9, and 12 hits, respectively. This suggests that these models impose more refined spatial and functional constraints that may better recognize specific features relevant to potent Na_V_1.7 inhibition. **LBPM_Na**_**V**_**1.7_Cluster4** and _**Cluster5** consistently identified compounds that featured an acylsulfonamide group, while **LBPM_Na**_**V**_**1.7_Cluster 9** and **_Cluster 10** retrieved arylsulfonamide derivatives. Overall, these findings highlight the importance of both pharmacophore diversity and careful model refinement in virtual screening workflows. The findings also suggest that specific pharmacophore models may offer unique advantages, either in targeting defined chemical classes or in optimizing isoform selectivity.

### Selectivity prediction of Na_V_1.7 inhibitors against Na_V_1.5 LBPMs

In addition to activity profiling, pharmacophore models can be used to predict target selectivity [41]. Given that Na_V_1.5 inhibition is associated with cardiotoxicity, particularly with long-term treatments, we investigated whether the selectivity of Na_V_1.7 inhibitors over the Na_V_1.5 channel could be assessed by screening the inhibitor set against Na_V_1.5 LBPMs.

To construct the Na_V_1.5 pharmacophore models, we followed the same approach as for Na_V_1.7. The Na_V_1.5 inhibitor dataset, containing 188 compounds, was first clustered based on pharmacophore RDF-code similarity, resulting in 22 clusters. Of these, nine clusters contained more than five ligands and were selected for LBPM construction (Table 2, Fig. S6). Following validation using both active compounds and decoys, **LBPM_Na**_**V**_**1.5_Cluster22** was found to be less selective. Consequently, we re-clustered the molecules in cluster 22 using pharmacophore alignment scores as the similarity metric, applying default parameters. This refinement yielded eight new clusters, four of which contained more than five molecules. These four clusters were subsequently used to generate additional Na_V_1.5 LBPMs (Table 2, Fig. S7).

After validating the Na_V_1.5 LBPMs, we used them to screen the Na_V_1.7 inhibitor libraries (Table 2). Most of the models identified several Na_V_1.7 inhibitors. Upon re-examining activity data from the VGSC database [29], we confirmed that many potent Na_V_1.7 inhibitors also exhibit inhibitory activity against Na_V_1.5, although typically at higher concentrations. This was further demonstrated by the detailed analysis of compounds retrieved from clusters **LBPM_Na**_**V**_**1.5_Cluster13** and **_Cluster17**. All investigated molecules were shown to be acylsulfonamide derivatives exhibiting inhibitory activity in the low to medium nanomolar range against Na_V_1.7 channels [42–44], and of these, only two compounds displayed subnanomolar potency [16, 45]. Except for two molecules for which no activity data against the Na_V_1.5 isoform were reported, all compounds showed an inhibitory effect on Na_V_1.5 channels, albeit at higher concentrations, typically in the low micromolar range (IC_50_= 8.7–22 µM) [42–44]. Notably, the two subnanomolar Na_V_1.7 inhibitors showed low nanomolar potency also against Na_V_1.5 isoform (IC_50_~50 nM) [16, 45]. These findings illustrate the challenges of using LBPMs to discriminate between closely related sodium channel isoforms, particularly when compounds share a common sulfonamide scaffold and retain measurable activity across multiple Na_V_ subtypes. The observed overlap between the Na_V_1.7 and Na_V_1.5 pharmacophore models likely reflects the high structural conservation of the VSD-IV binding site, where key interactions with gating-charge arginine residues and surrounding hydrophobic regions are preserved across isoforms. Consequently, many pharmacophoric features associated with potent Na_V_1.7 inhibition are also compatible with Na_V_1.5 binding. While the Na_V_1.5 LBPMs provided useful information for prioritizing compounds with potentially reduced Na_V_1.5 liability, the results indicate that ligand-based pharmacophore screening alone is insufficient for reliable prediction of subtype selectivity within the sulfonamide inhibitor class. Additional structure-based and experimental approaches are therefore required to accurately assess and optimize isoform selectivity.

**Table 2:** Hit rates of virtual screening using Na_V_1.5 pharmacophore models and Na_V_1.7 inhibitor libraries

	Nav1.7 IC_50_ ≤ 10 nM	Nav1.7 IC_50_ ≤ 100 nM	Nav1.7 all
	205 inhibitors	605 inhibitors	1512 inhibitors
LBPM_Nav1.5_Cluster13	2 hits	4 hits	4 hits
LBPM_Nav1.5_Cluster15	1 hit	11 hits	34 hits
LBPM_Nav1.5_Cluster16	7 hits	8 hits	35 hits
LBPM_Nav1.5_Cluster17	0 hits	2 hits	5 hits
LBPM_Nav1.5_Cluster18	10 hits	19 hits	154 hits
LBPM_Nav1.5_Cluster19	24 hits	106 hits	609 hits
LBPM_Nav1.5_Cluster20	42 hits	113 hits	202 hits
LBPM_Nav1.5_Cluster21	6 hits	22 hits	52 hits
LBPM_Nav1.5_Cluster22	202 hits	588 hits	1466 hits
LBPM_Nav1.5_Cluster22_5	56 hits	114 hits	234 hits
LBPM_Nav1.5_Cluster22_6	27 hits	64 hits	153 hits
LBPM_Nav1.5_Cluster22_7	32 hits	69 hits	134 hits
LBPM_Nav1.5_Cluster22_8	0 hits	3 hits	32 hits

### Use of Na_V_1.7 LBPMs in virtual screening to identify novel inhibitors

A series of sulfonamide-based Na_V_ inhibitors, not included in the VGSC database [29], was collected from recent literature [17, 18, 46–50]. To assess whether our Na_V_1.7-focused LBPMs could be utilized to identify novel inhibitors, this set of 22 compounds was employed as an external validation library for virtual screening. LBPMs were able to identify three inhibitors from this dataset, highlighting their potential for discovery of novel Na_V_1.7 inhibitors.

To further investigate the performance of each individual LBPM, a detailed analysis of the virtual screening results was performed by mapping the retrieved hits. The number of matched compounds varied across the models, reflecting the structural diversity encoded by the training ligands used to generate each pharmacophore. All retrieved molecules proved to be effective Na_V_1.7 inhibitors, showing activity in the micromolar range. Notably, **LBPM_Na**_**V**_**1.7_Cluster12_3** retrieved the highest number of hits (*n* = 2), *14f* and *14 g* (Fig. S8), both of them featuring an arylsulfonamide group [17]. Although these compounds were designed and demonstrated to be selective Na_V_1.3 inhibitors (*14f*, IC_50_, inactivated-state Na_V_1.3 current = 0.38 µM; *14 g*, IC_50_, inactivated-state Na_V_1.3 current = 0.44 µM), they also exhibited significant inhibitory activity on the inactivated state of Na_V_1.7 channels (*14f*, IC_50_= 8.4 µM; *14 g*, IC_50_= 9.31 µM), thereby confirming the capability of our model to identify Na_V_1.7 inhibitors [17]. **LBPM_Na**_**V**_**1.7_Cluster10** retrieved 1 hit, the arylsulfonamide derivative *29* (Fig. S8, Supplementary Information), that was proven to be a potent Na_V_1.7 inhibitor (IC_50_ = 0.019 µM) [47]. Most models failed to identify any compounds from the external validation set, suggesting that their pharmacophoric constraints may be too restrictive to detect novel chemotypes, particularly when applied to scaffolds not present in the original training set. Overall, the differential retrieval capacity of the LBPMs underscores the relevance of pharmacophore diversity in capturing distinct binding motifs and suggests that specific models may be better suited for identifying certain subclasses of Na_V_1.7 inhibitors.

Finally, we conducted virtual screening using the Na_V_1.7 LBPMs against a library of 3 million commercially available compounds. As expected, the feature-rich LBPMs, which previously identified only a small number of Na_V_1.7 actives from the known dataset and demonstrated strong discrimination between actives and decoys, also retrieved a low number of hits in this screening (Table 1). However, in this context, a low hit rate is desirable, as compounds that match these highly specific pharmacophore models are more likely to exhibit true on-target activity. Therefore, we focused on virtual screening hits retrieved by **LBPM_Na**_**V**_**1.7_Cluster4** (13 hits), **_Cluster9**, **_Cluster12.6**, **_Cluster12.8** and **_Cluster13** (1 hit each) and in addition **LBPM_Na**_**V**_**1.7_Cluster8** (519 hits). All hit compounds were docked to the Na_V_1.7 DIV binding site (PDB entry: 8F0S [18]) and after VS results examination, 10 molecules were selected for biological evaluation via patch-clamp electrophysiology on human Na_V_1.7 (Table 3 and Fig. S9). Docking scores were used solely as a prioritization criterion and qualitative assessment of binding plausibility. No direct relationship between docking score and experimental potency was assumed. Overall, the binding mode of the known inhibitor was well reproduced (RMSD 1.95 Å), involving stabilizing interactions with most amino acid residues in the binding site [18].

The compounds retrieved by **LBPM_Na**_**V**_**1.7_Cluster4** were small molecules predominantly featuring bicyclic nitrogen-containing aromatic ring systems and, in several cases, sulfonamide moieties that were predicted to form interactions with arginine residues. The top-ranked compound, a small dimethylbenzenesulfonamide derivative **1** (Table 3 and Fig. S9), was selected for biological evaluation. **LBPM_Na**_**V**_**1.7_Cluster9**, **_Cluster12_8** and **_Cluster13** each yielded a single retrieved hit, all of which were selected for biological testing. Compound **2** retrieved from **LBPM_Na**_**V**_**1.7_Cluster9** was a small benzenesulfonamide derivative, which was also predicted to be selective against the Na_V_1.5 isoform (Table 3). On the other hand, **LBPM_Na**_**V**_**1.7_Cluster12_8** and **_Cluster12_13** retrieved larger molecules. Specifically, **LBPM_Na**_**V**_**1.7_Cluster12_8** identified a sulfathiazole derivative **3**, an extensively validated scaffold for Na_V_1.7 inhibition, known to form crucial interactions with VSD-IV arginine residues [14]. **LBPM_Na**_**V**_**1.7_Cluster13** identified a pyrimidine derivative **4** lacking the typical sulfonamide moiety.

**LBPM_Na**_**V**_**1.7_Cluster8** retrieved 519 hits from the commercially available compound library. The predicted binding modes within the Na_V_1.7 DIV binding site of the 200 highest-ranked compounds were visually inspected, and six molecules (**5–10**, Table 3 and Fig. S9) were selected for biological evaluation. As aryl sulfonamide derivatives, these molecules were predicted to form stabilizing interactions with the arginine “gating charge” residues, consistent with the established sulfonamide-based inhibitors binding poses [14, 18]. Hydrophobic interactions with Ile and Leu residues contributed to the stabilization of the ligand-protein complexes.

Notably, fourteen of the inspected virtual screening hits were identified as known Na_V_ channel inhibitors. The two top-ranked known molecules are presented in Table 4 and Fig. S10 (**11** and **12**) [42, 51, 52], while the other twelve inhibitors, with reported inhibitory concentrations ≤ 25.0 µM [52], are presented in Table S1 (Supplementary Information). Interestingly, both **LBPM_Na**_**V**_**1.7_Cluster12_6** and _**Cluster8** identified a known aryl sulfonamide-based Na_V_1.7 inhibitor (**11**), which had an estimated IC_50_ of 1.1 nM [42, 51]. This result confirms the predictive ability of these models for identifying high-affinity Na_V_1.7 ligands and indicates that they likely contain the required key features for potent Na_V_1.7 inhibition.

**Table 3:** Ten hits selected for biological evaluation based on the virtual screening performed using the Na_V_1.7 ligand-based pharmacophore models (LBPMs) against a library of commercially available compounds: their alignment with the Na_V_1.7 LBPM, docking binding mode in the Na_V_1.7 DIV binding site and predicted selectivity over Na_V_1.5

Comp.	Alignment of the VS hit with LBPM	Docking binding mode of the VS hit	Predicted Na_V_1.5 selectivity
1	LBPM_Nav1.7_Cluster4		No
2	LBPM_Nav1.7_Cluster9		Yes
3	LBPM_Nav1.7_Cluster12_8		No
4	LBPM_Nav1.7_Cluster13		No
5	LBPM_Nav1.7_Cluster8		Yes
6	LBPM_Nav1.7_Cluster8		No
7	LBPM_Nav1.7_Cluster8		No
8	LBPM_Nav1.7_Cluster8		No
9	LBPM_Nav1.7_Cluster8		Yes
10	LBPM_Nav1.7_Cluster8		No

**Table 4:** The two top-ranked known Na_V_1.7 inhibitors identified with virtual screening performed using the Na_V_1.7 ligand-based pharmacophore models (LBPMs) against a library of commercially available compounds: their alignment with the Na_V_1.7 LBPM, docking binding mode in the Na_V_1.7 DIV binding site and predicted selectivity over Na_V_1.5

Comp.	Alignment of the VS hit with LBPM	Docking binding mode of the VS hit	Predicted Na_V_1.5 selectivity
11	LBPM_Nav17_Cluster12_6		No
12	LBPM_Nav1.7_Cluster8		No

### In vitro evaluation of Na_V_1.7 inhibition and subtype selectivity

**Fig. 2:**
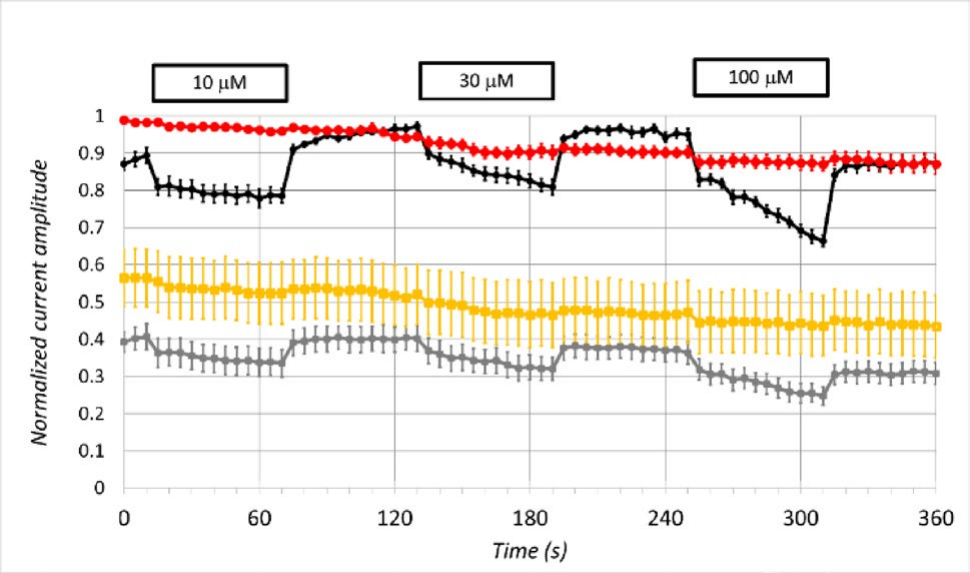
Average of normalized current traces demonstrating the selective Na_V_1.7 inhibition (*n* = 8) by compound **3** compared with Na_V_1.5 (*n* = 6). Error bars represent ± SEM. The compound concentrations applied are represented above the graph, and the application duration is represented by the width of the text box border (60 s). Black (resting channel inhibition) and grey (inactivated channel inhibition) traces represent the compound effect on the Na_V_1.7 amplitudes over time. Red (resting channel inhibition) and orange (inactivated channel inhibition) traces represent the compound effect on the Na_V_1.5 amplitudes

Ten virtual screening hits were tested on hNa_V_1.7. Out of the tested set, seven compounds did not produce any detectable inhibition within the investigated concentration range (Fig. S11). However, hit compounds **1**, **3**, and **9** demonstrated reproducible and quantifiable inhibition. Subsequent evaluation against the cardiac hNa_V_1.5 isoform revealed varying degrees of subtype selectivity. Among the tested compounds, compound **3** emerged as the only compound exhibiting pronounced selectivity for Na_V_1.7 over Na_V_1.5 (Fig. 2). In contrast, compound **1** showed moderate yet recognizable inhibition of Na_V_1.5, while compound **9** exhibited comparable inhibitory activity on both isoforms, lacking the desired target selectivity (Fig. S12).

### Kinetic characterization and state-dependent binding of compound 3

Determining an exact IC_50_ value for compound **3** proved challenging due to its exceptionally slow binding kinetics within the microfluidic system. During the standard 60-second compound application window, a steady-state block was not achieved at 30 µM. At 100 µM, the current amplitude exhibited a continuous, non-exponential decline, producing at least 30% inhibition of the hNa_V_1.7 current. Following a 60-second washout period, the block was found to be partially irreversible, with approximately 10% residual inhibition remaining or washing out slowly. In contrast, the application of 100 µM of compound **3** to hNa_V_1.5 resulted in only marginal inhibition (a few percent) that was fully reversible, confirming that **3** is at least one order of magnitude more effective on Na_V_1.7 (Fig. 2). The tested molecules did not exhibit a strong preference for the inactivated state over the resting state. As for compound **3**, the inhibition measured from the resting state was not significantly different from the inhibition measured following the inactivating prepulse (both yielding approximately 30% normalized fractional block at the highest concentrations). Compounds **1** and **9** similarly showed no distinct state-dependent block under the applied protocol (Fig. S13). Future studies involving extended incubation protocols, kinetic modeling, and full concentration-response characterization will be required to establish precise potency estimates and elucidate the molecular mechanism of inhibition.

## Conclusion

The present study has employed a LBPM approach to develop and validate predictive models for discovering novel Na_V_1.7 inhibitors. Starting from a curated VGSC database [29] focused on potent sulfonamide-based compounds (IC_50_ ≤ 10 nM), we successfully generated 14 pharmacophore models. The most selective models, including **LBPM_Na**_**V**_**1.7_Cluster4**, **_Cluster9**, and **_Cluster13**, demonstrated excellent discrimination between active compounds and decoys, achieving high AUC values and strong enrichment factors. Screening the Na_V_1.7 LBPMs against inhibitor libraries of other Na_V_1.x isoforms revealed varying degrees of specificity, highlighting an overall challenge in achieving selectivity, particularly with a single pharmacophore model, given the high sequence similarity between isoforms. The development of Na_V_1.5 LBPMs provided preliminary information regarding potential Na_V_1.5 liability, although reliable selectivity prediction remains challenging and requires complementary approaches. Screening against 3 million commercially available compounds confirmed their utility, retrieving both hits suitable for experimental validation and known Na_V_1.7 inhibitors. Docking studies supported predicted binding modes and the experimental validation demonstrates that the workflow can identify compounds with measurable Na_V_1.7 inhibitory activity and provides useful starting points for further optimization. In summary, this work underscores the value of ligand-based drug design in the early stages of Na_V_1.7 drug discovery, providing novel validated tools that can be used for the identification of Na_V_1.7 inhibitors. However, only a limited number of compounds could be experimentally evaluated, and therefore the reported hit rate should be interpreted as a preliminary assessment of workflow performance rather than a definitive measure of predictive power. The novel hit scaffolds identified here can provide new starting points for lead optimization and our approach may help expand the structural diversity of Na_V_1.7 inhibitors beyond the current canonical scaffolds.

## Supplementary Information

Below is the link to the electronic supplementary material.

Supplementary Material 1

## Data Availability

The data that support the findings of this study are available from the corresponding author.

## Abbreviations

ADME: Absorption, distribution, metabolism, and excretion
AR: Aromatic ring
AUC: Area under the curve
CADD: Computer aided drug design
CIDs: Compound identifiers
CNS: Central nervous system
DI-DIV: Domains I-IV
DUD-E: Database of useful decoys-enhanced
EF: Enrichment factor
HAL: Halogen bond
HBA: Hydrogen bond acceptor
HBD: Hydrogen bond donor
H: Hydrophobic
IC_50_: Half maximal inhibitory concentration
IUPAC: International union of pure and applied chemistry
LBPMs: Ligand based pharmacophore models
Na_V_: Voltage-gated sodium channels
PDB: Protein data bank
PNS: Peripheral nervous system
ROC: Receiver operating characteristic
SAR: Structure activity relationship
SMILES: Simplified molecular-input line-entry system
VSD-IV: Voltage-sensing domain IV
VGSC: Voltage gated sodium channel
