# Supplementary material for: Ligand-based pharmacophore modeling for the discovery of NaV1.7 inhibitors
Source: J Comput Aided Mol Des. 2026 Aug 7;40(1):198. doi: 10.1007/s10822-026-00898-z (PMC13447489; doi:10.1007/s10822-026-00898-z)
Supplement: Supplementary file 1 [file 10822_2026_898_MOESM1_ESM.docx]

SUPPLEMENTARY INFORMATION

**Ligand-based pharmacophore modeling for the discovery of Na_V_1.7 inhibitors**

Martina Piga ^a^, Peter Lukacs ^b^, Krisztina Pesti ^b,c^, György Várady ^d^, Zoltan Varga ^e^_,_ Nace Zidar ^a^, Tihomir Tomašič ^a,^*

*^a^ Faculty of Pharmacy, University of Ljubljana, Aškerčeva cesta 7, Ljubljana 1000, Slovenia*

*^b^ Agricultural Institute, Centre for Agricultural Research, Martonvásár, Hungary*

*^c^ HUN-REN Institute of Experimental Medicine, Budapest, Hungary*

*^d^* *Institute of Molecular Life Sciences, HUN-REN Research Centre for Natural Sciences, Budapest, Hungary*

*^e^ Department of Biophysics and Cell Biology, Faculty of Medicine, University of Debrecen, Egyetem ter1, 4032 Debrecen, Hungary*

*Corresponding author: E-mail address: [tihomir.tomasic@ffa.uni-lj.si](mailto:tihomir.tomasic@ffa.uni-lj.si)

**Contents:**

**Figure S1.** Voltage protocol designed for testing activation and inactivation parameters of compound effects in 5 s resolution...………………………………………….………………………...…………S4

**Figure S2.** Ligand-based pharmacophore models for Na_V_1.7 inhibitors with IC_50_≤10 nM… **A**) LBPM_Nav1.7_Cluster5, **B**) LBPM_Nav1.7_Cluster8, **C**) LBPM_Nav1.7_Cluster10, **D**) LBPM_Nav1.7_Cluster11, and **E**) LBPM_Nav1.7_Cluster12. Each model is aligned with the inhibitor with the highest pharmacophore fit score. The pharmacophore features are as follows: hydrophobic (H, yellow spheres), aromatic (AR, blue disc), hydrogen bond donor (HBD, green arrow), and hydrogen bond acceptor (HBA, red sphere)…………………………………………………………...…………S4

**Figure S3.** ROC plot (curve shown in blue) generated from virtual screening of actives and generated decoys with **A**) LBPM_Nav1.7_Cluster4; **B**) LBPM_Nav1.7_Cluster5; **C**) LBPM_Nav1.7_Cluster8; **D**) LBPM_Nav1.7_Cluster9; **E**) LBPM_Nav1.7_Cluster10; **F**) LBPM_Nav1.7_Cluster11; **G**) LBPM_Nav1.7_Cluster12; **H**) LBPM_Nav1.7_Cluster13; TP = true positives; FP = false positives; AUC = area under the curve; EF = enrichment factor…………………..…………………………………………………………………...………….S6

**Figure S4.** Ligand-based pharmacophore models for Na_V_1.7 inhibitors with IC_50_≤10 nM. **A**) LBPM_Nav1.7_Cluster12_3; **B**) LBPM_Nav1.7_Cluster12_4; **C**) LBPM_Nav1.7_Cluster12_5; **D**) LBPM_Nav1.7_Cluster12_6; **E**) LBPM_Nav1.7_Cluster12_7; **F**) LBPM_Nav17_Cluster12_8. Each model is aligned with the inhibitor with the highest pharmacophore fit score. The pharmacophore features are as follows: hydrophobic (H, yellow spheres), aromatic (AR, blue disc), hydrogen bond donor (HBD, green arrow), and hydrogen bond acceptor (HBA, red sphere)..………………………...S7

**Figure S5.** ROC plot (curve shown in blue) generated from virtual screening of actives and generated decoys with **A**) LBPM_Nav1.7_Cluster12_3; **B**) LBPM_Nav1.7_Cluster12_4; **C**) LBPM_Nav1.7_Cluster12_5; **D**) LBPM_Nav1.7_Cluster12_6; **E**) LBPM_Nav1.7_Cluster12_7; **F**) LBPM_Nav1.7_Cluster12_8; TP = true positives; FP = false positives; AUC = area under the curve; EF = enrichment factor..………………………………………………………………..…………...……..S9

**Table S1.** Validation metrics for all Na_V_1.7 pharmacophore models………………………S10

**Figure S6.** Ligand-based pharmacophore models for Na_V_1.5 inhibitors with IC_50_≤10 nM. **A**) LBPM_Nav1.5_Cluster13; **B**) LBPM_Nav1.5_Cluster15; **C**) LBPM_Nav1.5_Cluster16; **D**) LBPM_Nav1.5_Cluster17; **E**) LBPM_Nav1.5_Cluster18; **F**) LBPM_Nav1.5_Cluster19, **G**) LBPM_Nav1.5_Cluster20; **H**) LBPM_Nav1.5_Cluster21; **I**) LBPM_Nav1.5_Cluster22_5; **J**) LBPM_Nav1.5_Cluster22_6; **K**) LBPM_Nav1.5_Cluster22_7; **L**) LBPM_Nav1.5_Cluster22_8. Each model is aligned with the inhibitor with the highest pharmacophore fit score. The pharmacophore features are as follows: hydrophobic (H, yellow spheres), aromatic (AR, blue disc), hydrogen bond donor (HBD, green arrow), and hydrogen bond acceptor (HBA, red sphere)..…………………..…...S11

**Figure S7.** ROC plot (curve shown in blue) generated from virtual screening of actives and generated decoys with **A**) LBPM_Nav1.5_Cluster13; **B**) LBPM_Nav1.5_Cluster15; **C**) LBPM_Nav1.5_Cluster16; **D**) LBPM_Nav1.5_Cluster17; **E**) LBPM_Nav1.5_Cluster18; **F**) LBPM_Nav1.5_Cluster19, **G**) LBPM_Nav1.5_Cluster20; **H**) LBPM_Nav1.5_Cluster21; **I**) LBPM_Nav1.5_Cluster22_5; **J**) LBPM_Nav1.5_Cluster22_6; **K**) LBPM_Nav1.5_Cluster22_7; **L**) LBPM_Nav1.5_Cluster22_8; TP = true positives; FP = false positives; AUC = area under the curve; EF = enrichment factor..………………………………………………...…….………………...…...…...S13

**Figure S8.** Overlay of **A**) inhibitor *14f* and LBPM_Nav1.7_Cluster12_3; **B**) inhibitor *14g* and LBPM_Nav1.7_Cluster12_3; **C**) inhibitor *29* and LBPM_Nav1.7_Cluster10. The pharmacophore features are as follows: hydrophobic (H, yellow spheres), aromatic (AR, blue disc), hydrogen bond donor (HBD, green arrow), hydrogen bond acceptor (HBA, red sphere), and (HAL, dotted pink arrow)....……………………………………………………………………………….……………..S16

**Figure S9****.** Overlay of **A**) compound **1** and LBPM_Nav1.7_Cluster4; **B**) compound **2** and LBPM_Nav1.7_Cluster9; **C**) compound **3** and LBPM_Nav1.7_Cluster12_8; **D**) compound **4** and LBPM_Nav1.7_Cluster13; **E-J**) compounds **5-10** and LBPM_Nav1.7_Cluster8. The pharmacophore features are as follows: hydrophobic (H, yellow spheres), aromatic (AR, blue disc), hydrogen bond donor (HBD, green arrow), hydrogen bond acceptor (HBA, red sphere), and (HAL, dotted pink arrow)...………………………………………………………………………….…………………...S17

**Figure S10.** Overlay of **A**) compound **11** and LBPM_Nav1.7_Cluster12_6; **B**) compound 1**2** and LBPM_Nav1.7_Cluster8. The pharmacophore features are as follows: hydrophobic (H, yellow spheres), aromatic (AR, blue disc), hydrogen bond donor (HBD, green arrow), hydrogen bond acceptor (HBA, red sphere), and (HAL, dotted pink arrow)....………………………………………………………………………………...…………....S18

**Table S1.** Known Na_V_1.7 inhibitors identified with virtual screening performed using the Na_V_1.7 ligand-based pharmacophore models (LBPMs) against a library of commercially available compounds: their alignment with the Na_V_1.7 LBPM and docking binding mode in the Na_V_1.7 DIV binding site...……………………………………………………………………………………………..…...S19

**Figure S11.** Dot plot of inhibition levels on NaV1.7 for compounds 1–10. Event 21 (A) represents inhibition in the resting state, whereas event 47 (B) shows the inactivated state inhibition...........…...S23

**Figure S12.** Data illustrating the non-selective (equipotent) effects of compounds 1 (A) and 9 (B) on both Na_V_1.7 and Na_V_1.5. Black (resting channel inhibition; n = 5) and grey (inactivated channel inhibition; n = 6) traces represent the compound effects on the Na_V_1.7 amplitudes over time. Red (resting channel inhibition; n = 7) and orange (inactivated channel inhibition; n = 7) traces represent the compound effects on the Na_V_1.5 amplitudes. Error bars represent ± SEM.......................…………....S25

**Figure S1.** Voltage protocol designed for testing activation and inactivation parameters of compound effects in 5 s resolution.

**A** **B**

**C** **D**

**E**

**Figure S2.** Ligand-based pharmacophore models for Na_V_1.7 inhibitors with IC_50_≤10 nM. **A**) LBPM_Nav1.7_Cluster5, **B**) LBPM_Nav1.7_Cluster8, **C**) LBPM_Nav1.7_Cluster10, **D**) LBPM_Nav1.7_Cluster11, and **E**) LBPM_Nav1.7_Cluster12. Each model is aligned with the inhibitor with the highest pharmacophore fit score. The pharmacophore features are as follows: hydrophobic (H, yellow spheres), aromatic (AR, blue disc), hydrogen bond donor (HBD, green arrow), and hydrogen bond acceptor (HBA, red sphere).

**A** **B**

**C** **D**

**E** **F** **G** **H**

**Figure S3.** ROC plot (curve shown in blue) generated from virtual screening of actives and generated decoys with **A**) LBPM_Nav1.7_Cluster4; **B**) LBPM_Nav1.7_Cluster5; **C**) LBPM_Nav1.7_Cluster8; **D**) LBPM_Nav1.7_Cluster9; **E**) LBPM_Nav1.7_Cluster10; **F**) LBPM_Nav1.7_Cluster11; **G**) LBPM_Nav1.7_Cluster12; **H**) LBPM_Nav1.7_Cluster13; TP = true positives; FP = false positives; AUC = area under the curve; EF = enrichment factor.

**A** **B**

**C** **D**

**E****F**

**Figure S4.** Ligand-based pharmacophore models for Na_V_1.7 inhibitors with IC_50_≤10 nM. **A**) LBPM_Nav1.7_Cluster12_3; **B**) LBPM_Nav1.7_Cluster12_4; **C**) LBPM_Nav1.7_Cluster12_5; **D**) LBPM_Nav1.7_Cluster12_6; **E**) LBPM_Nav1.7_Cluster12_7; **F**) LBPM_Nav17_Cluster12_8. Each model is aligned with the inhibitor with the highest pharmacophore fit score. The pharmacophore features are as follows: hydrophobic (H, yellow spheres), aromatic (AR, blue disc), hydrogen bond donor (HBD, green arrow), and hydrogen bond acceptor (HBA, red sphere).

**A** **B**

**C** **D**

**E** **F**

**Figure S5.** ROC plot (curve shown in blue) generated from virtual screening of actives and generated decoys with **A**) LBPM_Nav1.7_Cluster12_3; **B**) LBPM_Nav1.7_Cluster12_4; **C**) LBPM_Nav1.7_Cluster12_5; **D**) LBPM_Nav1.7_Cluster12_6; **E**) LBPM_Nav1.7_Cluster12_7; **F**) LBPM_Nav1.7_Cluster12_8; TP = true positives; FP = false positives; AUC = area under the curve; EF = enrichment factor.

**Table S1.** Validation metrics for all Na_V_1.7 pharmacophore models.

|  | EF  1% | EF  5% | AUC  1% | AUC  5% | sensitivity | specificity | Matthews correlation coefficient |
| --- | --- | --- | --- | --- | --- | --- | --- |
| LBPM_Nav1.7_Cluster4 | 2.7 | 2.7 | 1.00 | 0.64 | 1.4% | 100% | 0.090 |
| LBPM_Nav1.7_Cluster5 | 2.8 | 2.8 | 0.67 | 0.53 | 0.3% | 100% | 0.046 |
| LBPM_Nav1.7_Cluster8 | 2.8 | 2.8 | 1.00 | 1.00 | 9.9% | 100% | 0.257 |
| LBPM_Nav1.7_Cluster9 | 2.8 | 2.8 | 1.00 | 1.00 | 31.1% | 100% | 0.475 |
| LBPM_Nav1.7_Cluster10 | 2.8 | 2.1 | 1.00 | 1.00 | 8.3% | 98.5% | 0.166 |
| LBPM_Nav1.7_Cluster11 | 2.8 | 2.8 | 1.00 | 1.00 | 52.9% | 99.9% | 0.646 |
| LBPM_Nav1.7_Cluster12 | 2.0 | 2.6 | 0.63 | 0.91 | 88.8% | 86.6% | 0.736 |
| LBPM_Nav1.7_Cluster12_3 | 2.7 | 2.7 | 0.98 | 0.99 | 67.1% | 94.2% | 0.657 |
| LBPM_Nav1.7_Cluster12_4 | 2.8 | 2.8 | 1.00 | 0.85 | 3.5% | 100% | 0.151 |
| LBPM_Nav1.7_Cluster12_5 | 2.8 | 2.8 | 1.00 | 0.98 | 4.8% | 100% | 0.178 |
| LBPM_Nav1.7_Cluster12_6 | 2.8 | 2.8 | 1.00 | 1.00 | 9.9% | 100% | 0.257 |
| LBPM_Nav1.7_Cluster12_7 | 2.8 | 2.8 | 1.00 | 1.00 | 7.8% | 100% | 0.228 |
| LBPM_Nav1.7_Cluster12_8 | 2.8 | 2.8 | 1.00 | 1.00 | 31.2% | 100% | 0.475 |
| LBPM_Nav1.7_Cluster13 | 2.8 | 2.8 | 1.00 | 0.76 | 2.6% | 100% | 0.130 |

**A****B****C****D****E****F****G****H****I****J**

**K****L**

**Figure S6.** Ligand-based pharmacophore models for Na_V_1.5 inhibitors with IC_50_≤10 nM. **A**) LBPM_Nav1.5_Cluster13; **B**) LBPM_Nav1.5_Cluster15; **C**) LBPM_Nav1.5_Cluster16; **D**) LBPM_Nav1.5_Cluster17; **E**) LBPM_Nav1.5_Cluster18; **F**) LBPM_Nav1.5_Cluster19, **G**) LBPM_Nav1.5_Cluster20; **H**) LBPM_Nav1.5_Cluster21; **I**) LBPM_Nav1.5_Cluster22_5; **J**) LBPM_Nav1.5_Cluster22_6; **K**) LBPM_Nav1.5_Cluster22_7; **L**) LBPM_Nav1.5_Cluster22_8. Each model is aligned with the inhibitor with the highest pharmacophore fit score. The pharmacophore features are as follows: hydrophobic (H, yellow spheres), aromatic (AR, blue disc), hydrogen bond donor (HBD, green arrow), and hydrogen bond acceptor (HBA, red sphere).

**A** **B****C** **D****E** **F****G** **H****I** **J****K** **L**

**Figure S7.** ROC plot (curve shown in blue) generated from virtual screening of actives and generated decoys with **A**) LBPM_Nav1.5_Cluster13; **B**) LBPM_Nav1.5_Cluster15; **C**) LBPM_Nav1.5_Cluster16; **D**) LBPM_Nav1.5_Cluster17; **E**) LBPM_Nav1.5_Cluster18; **F**) LBPM_Nav1.5_Cluster19, **G**) LBPM_Nav1.5_Cluster20; **H**) LBPM_Nav1.5_Cluster21; **I**) LBPM_Nav1.5_Cluster22_5; **J**) LBPM_Nav1.5_Cluster22_6; **K**) LBPM_Nav1.5_Cluster22_7; **L**) LBPM_Nav1.5_Cluster22_8; TP = true positives; FP = false positives; AUC = area under the curve; EF = enrichment factor

**A**

**B**
**C**

**Figure S8.** Overlay of **A**) inhibitor *14f* and LBPM_Nav1.7_Cluster12_3; **B**) inhibitor *14g* and LBPM_Nav1.7_Cluster12_3; **C**) inhibitor *29* and LBPM_Nav1.7_Cluster10. The pharmacophore features are as follows: hydrophobic (H, yellow spheres), aromatic (AR, blue disc), hydrogen bond donor (HBD, green arrow), hydrogen bond acceptor (HBA, red sphere), and (HAL, dotted pink arrow).

**A****B**

**C****D**

**E****F**

**G****H**

**I****J**

**Figure S9.** Overlay of **A**) compound **1** and LBPM_Nav1.7_Cluster4; **B**) compound **2** and LBPM_Nav1.7_Cluster9; **C**) compound **3** and LBPM_Nav1.7_Cluster12_8; **D**) compound **4** and LBPM_Nav1.7_Cluster13; **E-J**) compounds **5-10** and LBPM_Nav1.7_Cluster8. The pharmacophore features are as follows: hydrophobic (H, yellow spheres), aromatic (AR, blue disc), hydrogen bond donor (HBD, green arrow), hydrogen bond acceptor (HBA, red sphere), and (HAL, dotted pink arrow).

**A****B**

**Figure S10.** Overlay of **A**) compound **11** and LBPM_Nav1.7_Cluster12_6; **B**) compound 1**2** and LBPM_Nav1.7_Cluster8. The pharmacophore features are as follows: hydrophobic (H, yellow spheres), aromatic (AR, blue disc), hydrogen bond donor (HBD, green arrow), hydrogen bond acceptor (HBA, red sphere), and (HAL, dotted pink arrow).

**Table S1.** Known Na_V_1.7 inhibitors identified with virtual screening performed using the Na_V_1.7 ligand-based pharmacophore models (LBPMs) against a library of commercially available compounds: their alignment with the Na_V_1.7 LBPM and docking binding mode in the Na_V_1.7 DIV binding site.

| Alignment of the VS hit with LBPM | Docking binding mode of the VS hit |
| --- | --- |
|   LBPM_Nav1.7_Cluster8 |  |
|   LBPM_Nav1.7_Cluster8 |  |
|   LBPM_Nav1.7_Cluster8 |  |
|   LBPM_Nav1.7_Cluster8 |  |
|   LBPM_Nav1.7_Cluster8 |  |
|   LBPM_Nav1.7_Cluster8 |  |
|   LBPM_Nav1.7_Cluster8 |  |
|   LBPM_Nav1.7_Cluster8 |  |
|   LBPM_Nav1.7_Cluster8 |  |
|   LBPM_Nav1.7_Cluster8 |  |
|   LBPM_Nav1.7_Cluster8 |  |
| LBPM_Nav17_Cluster8 |  |

**A**

**B**

**Figure S11.** Dot plot of inhibition levels on Na_V_1.7 for compounds **1–10**. Event 21 (**A**) represents inhibition in the resting state, whereas event 47 (**B**) shows the inactivated state inhibition.

**Figure S12.** Data illustrating the non-selective (equipotent) effects of compounds **1** (**A**) and **9** (**B**) on both Na_V_1.7 and Na_V_1.5. Black (resting channel inhibition; n = 5) and grey (inactivated channel inhibition; n = 6) traces represent the compound effects on the Na_V_1.7 amplitudes over time. Red (resting channel inhibition; n = 7) and orange (inactivated channel inhibition; n = 7) traces represent the compound effects on the Na_V_1.5 amplitudes. Error bars represent ± SEM.
